# Flourishing and its influencing factor in inflammatory bowel disease patients: a latent profile analysis

**DOI:** 10.3389/fpsyt.2026.1751497

**Published:** 2026-02-20

**Authors:** Bingyuan Lu, Linlin Ma, Fei Xia, Lu Zhang, Ying Liu, Xiaoyan Yu, Renjuan Sun, Yanfang Luo

**Affiliations:** 1 Department of Gastroenterology, Affiliated Hospital of Jiangnan University, Wuxi, China; 2 Wuxi School of Medicine, Jiangnan University, Wuxi, China

**Keywords:** flourishing, inflammatory bowel disease, latent profile analysis, nursing, psychological resilience, social support

## Abstract

**Background:**

Flourishing is a key positive psychological construct that has been linked to favorable health-related outcomes in patients with inflammatory bowel disease in prior research. However, current research often overlooks the variations in flourishing levels within this population, as well as the mechanisms through which flourishing interacts with disease progression.

**Objective:**

This study aimed to identify latent categories of flourishing among patients with inflammatory bowel disease and to analyze the potential influencing factors.

**Methods:**

This study employed a cross-sectional, descriptive exploratory design involving 316 patients diagnosed with inflammatory bowel disease. Data collection was carried out using a general information questionnaire, the Flourishing Scale (FS), the IBD Self-Efficacy Scale (IBD-SES), the Resilience Scale for Inflammatory Bowel Disease (RS-IBD), and the Social Support Rating Scale (SSRS). Latent profile analysis (LPA) was utilized to identify potential subgroups exhibiting flourishing, while multiple logistic regression analysis was conducted to evaluate the influencing factors.

**Results:**

The flourishing of individuals with inflammatory bowel disease was classified into three latent groups: the low flourishing-low support beneficiary group (*n* = 66, 20.9%), the moderate flourishing-optimism weakening group (*n* = 182, 57.6%), and the high flourishing-social dignity achievement group (*n* = 68, 21.5%). A multiple logistic regression analysis indicated that marital status, monthly household income, duration of diagnosis, managing stress and emotions, managing symptoms and disease, maintaining remission, and emotional regulation were significant predictors of the latent categories of flourishing among the participants (all *p* < 0.05).

**Conclusions:**

Patients with inflammatory bowel disease demonstrate three distinct latent categories of flourishing. Healthcare professionals should implement more accurate and targeted intervention measures based on the characteristics and influencing factors of different potential categories, in order to improve the flourishing levels of patients with inflammatory bowel disease.

## 1 Introduction

Inflammatory bowel disease (IBD) is an immune-mediated gastrointestinal disorder that includes Crohn’s disease (CD) and ulcerative colitis (UC). It is characterized by chronic, recurrent intestinal inflammation, a prolonged course with frequent relapses, and presents significant challenges in achieving a cure. Currently, there is no definitive cure for this condition (1, 2). The global incidence of IBD has been increasing, with China’s rate rising from 1.45 per 100,000 people in 1990 to 3.62, resulting in an estimated cumulative total of approximately 911,000 cases (3). In addition to intestinal symptoms, IBD imposes a considerable public health burden. Patients frequently experience extra-intestinal manifestations such as joint degeneration and osteoporosis, and many suffer from mental health issues (4). Research indicates that over 32.1% of IBD patients experience anxiety, 25.2% suffer from depression, and numerous individuals endure stigma, loneliness, and fear of recurrence associated with the disease (5, 6). These psychological challenges not only significantly impair a patients’ health-related quality of life and functional status, but also affect disease prognosis and escalate medical costs. This underscores the urgent need to explore comprehensive disease management strategies (7, 8).

Traditionally, the management of IBD has focused mainly on drug treatment and alleviation of intestinal symptoms. However, patients, doctors, and families often overlook the crucial role of mental health support in treatment and rehabilitation (9). Increasing evidence suggests that psychological intervention is of significant value, capable of reducing pain, alleviating negative emotions, and positively regulating patients’ mental health (10). For instance, higher psychological resilience is independently associated with lower anxiety levels in IBD patients, with each additional point in resilience score reducing anxiety severity (11, 12). Meanwhile, higher perceived stress significantly increases the risk of clinical relapse (13). These findings collectively suggest that flourishing, a core concept within the framework of positive psychology, may represent a significant focus for understanding the psychological adaptation of patients with IBD. Broadly, flourishing is associated with positive outcomes for individuals with chronic diseases, including reduced self-reported negative emotions, enhanced subjective well-being, and improved self-management behaviors, all of which contribute to an overall enhancement in quality of life. It is important to explicitly note that the operationalization of flourishing in the present study is somewhat limited. We employed a self-assessment scale comprising eight items to evaluate participants’ subjective perception of flourishing, rather than conducting a comprehensive assessment of psychological functions and behaviors.

Flourishing is a comprehensive mental health state rooted in positive psychology theory, integrating emotional, psychological and social dimensions of health performance. It serves as an emerging, holistic and structured indicator for assessing subjective well-being (14). The founder of positive psychology, Martin Seligman, proposed in 2011 that the central theme of positive psychology is happiness, with its ultimate goal being the enhancement of individuals’ flourishing levels (15). Unlike previous mental health research (16)that primarily focused on minimizing negative emotions, positive psychology emphasizes the exploration of individual positive emotional experiences, thereby facilitating the development of positive psychological qualities and behavioral models. Studies have indicated that promoting flourishing is a key objective in the treatment of chronic diseases (17). For patients with IBD, clinically, flourishing can serve as an active psychological resource. It buffers the stress response pathway by promoting adaptive strategies such as problem-solving and seeking social support, thereby potentially alleviating the psychological burden caused by chronic inflammation and recurrence of disease symptoms (18). Moreover, patients with IBD who have a higher level of flourishing are more likely to view the disease as a controllable challenge, have a lower perception of the disease’s impact, and thus reduce its interference with daily functioning, social participation, and self-identity (19). At the same time, flourishing integrates core dimensions such as emotional health, sense of meaning in life, and social connection, which can partially counteract fatigue related to IBD, social isolation, and treatment uncertainty, and become an important protective factor for HRQoL (20).

In conclusion, despite the theoretical potential of flourishing, current research in this area exhibits significant deficiencies. The flourishing theory posits that the formation and maintenance of a flourishing state are driven by both an individual’s internal psychological resources and the external social environment. The former emphasizes positive psychological assets such as self-efficacy and psychological resilience, while the latter highlights nurturing factors from the environment, including social support. (21) However, existing studies have notable gaps in addressing this theoretical framework in the context of patients with IBD: on one hand, there is a lack of empirical data regarding the flourishing status of patients with IBD; on the other hand, existing studies have not adequately considered the differences in flourishing levels among patient groups, nor have they not fully elucidated the disparities in flourishing among patients with varying characteristics. Furthermore, self-efficacy, psychological resilience and social support, as key psychological and social factors influencing flourishing, have not been adequately incorporated into the analysis of the diversity of flourishing among patients with IBD.

Self-efficacy, a concept proposed by Bandura (22), refers to an individual’s belief in their ability to perform specific behaviors to achieve desired outcomes. This concept has been extensively applied in the health domain and is regarded as a positive psychological factor that may protect patients with IBD as they cope with and manage various challenges (23). The chronic and recurrent nature of gastrointestinal inflammation coupled with fluctuating symptoms during active phases of the disease, significantly impacts IBD patients’ perceptions of disease severity and stress levels, consequently increasing their psychological distress. Existing studies have confirmed that the prevalence of mental health comorbidities among IBD patients is significantly higher than that in the general population, with estimates suggesting rates that are approximately two to three times greater (24, 25). Moreover, research indicates that high self-efficacy among patients is closely associated with improved emotional regulation, reduced stress levels, and greater utilization of adaptive coping strategies (26). For IBD patients, the unpredictability of disease progression and the potential for social stigmatization of symptoms underscore the critical role of self-efficacy in effective disease management. Nonetheless, the relationship between self-efficacy and flourishing remains ambiguous and warrants further investigation.

Psychological resilience is a positive psychological trait that refers to an individual’s ability to protect or recover from adversity, threats, or significant pressures. This trait facilitates the restoration of normal physiological and psychological functions (27). Relevant studies indicate that individuals with high psychological resilience are more adept at integrating their positive psychological qualities with external support resources. By actively adapting to challenging situations and engaging in diverse health-promoting behaviors to address disease-related challenges, these individuals can effectively enhance and sustain their subjective well-being as well as their physical and mental health (28). It is important to note that the chronic persistence, symptom recurrence, and social stigmatization characteristics of IBD (29) make patients susceptible to developing post-traumatic stress disorder and predispose them to gastrointestinal stress sensitivity (30). This characteristic underscores the vital role of psychological resilience in mitigating disease-related stress and improving emotional health, while also serving as a significant factor influencing the flourishing of patients.

Social support refers to the perceived or actual assistance individuals receive from social interactions (31), and is primarily categorized into objective support, subjective support, and the degree of utilization of support. Living with IBD can be a stressful experience for patients. According to stress and coping theory, social support serves as an external resource that mitigates stress (32). As a protective resource, it alleviates emotional distress and empowers patients to actively manage their condition, thereby influencing their overall physical and mental well-being. Consequently, examining the impact of varying levels of social support on the flourishing of IBD patients can provide valuable insights into their needs and inform the development of targeted intervention strategies.

Previous studies have demonstrated that socio-demographic factors (such as marital status and economic burden) and disease-related factors (including disease duration, disease activity, and complications) are closely associated with the psychological outcomes of patients with IBD. Specifically, marital status may serve a buffering role; unmarried patients are more likely to experience negative psychological states due to a limited support network when confronted with the long-term uncertainties of their condition (33). Additionally, patients with low family income or those responsible for their own medical expenses often face a higher economic burden, which can increase their susceptibility to anxiety and emotional distress, thereby adversely affecting their mental health (34). Furthermore, patients diagnosed for less than one year and experiencing active disease must navigate both the uncertainty of prognosis and the pressures of role adaptation, which may hinder the development of a positive psychological state (35). The study also indicates that patients with IBD-related complications may endure greater physical and psychological pain, leading to a heavier emotional burden that is detrimental to the formation of a positive psychological state (36). Consequently, this study simultaneously examines these demographic and disease-related variables to identify the key factors influencing the psychological profile of IBD patients.

Latent Profile Analysis (LPA) is a person-centered approach that categorizes individuals into subgroups based on intricate patterns of variables, thereby facilitating the identification of potential heterogeneity in the literature (37). It has been extensively utilized in psychological research, including the examination of subtypes of psychological adaptation patterns (36), and the classification of disease burden subtypes in IBD patients (34). Therefore, this study focuses on IBD patients and employs latent profile analysis (LPA) to explore the heterogeneity of their flourishing. The primary objective is to identify the sociodemographic and clinical characteristics associated with these features and to analyze their relationship with self-efficacy, psychological resilience and social support. The research findings will provide a theoretical foundation for healthcare professionals to design targeted psychological support strategies, optimize disease management based on psychological factors, and develop personalized mental health plans. Ultimately, these efforts will enhance psychological rehabilitation outcomes and improve the quality of life for patients with IBD.

## 2 Materials and methods

### 2.1 Sampling and inclusion criteria

This cross-sectional exploratory survey was conducted at a tertiary hospital in Wuxi, China, involving a total of 316 individuals diagnosed with IBD between April and August 2025.

The inclusion criteria for participants were as follows: (1) aged 18 years or older, (2) met the diagnostic criteria for IBD (38), (3) demonstrated the ability to accurately read and comprehend the questionnaire without communication barriers, and (4) provided informed consent voluntarily. The exclusion criteria included: (1) a disease duration of less than three months, (2) life-threatening conditions confirmed by the attending physician via the electronic medical record include: severe organ failure and advanced malignant tumors. (3) additionally, severe mental or cognitive disorders are identified according to the Diagnostic and Statistical Manual of Mental Disorders (Fifth Edition), characterized by a score of ≤ 23 on the Mini-Mental State Examination Scale and a score of > 11 on the Hamilton Anxiety and Depression Scale. (39). The sample size was estimated based on the rule of thumb for multivariate analyses, which required 5–10 observations per independent variable (40). This study included a total of 27 independent variables; therefore, considering a 10% non-response rate, a minimum of 300 participants was deemed necessary. Meanwhile, with reference to Ferguson et al. (41), who recommended a minimum sample size of 300 for LPA, we ultimately enrolled 316 IBD patients.

### 2.2 Data collection

Two trained researchers conducted the survey with participants who met the inclusion criteria at the time of enrollment. Prior to administering the survey, the researchers provided a comprehensive explanation of the study’s purpose and obtained written informed consent from all participants. Patients were instructed to complete the anonymous questionnaire independently. Standardized instructions were provided to ensure clarity, encompassing the content, key points, and responses to potential questions. Fully completed questionnaires were collected for immediate quality control and any missing items were promptly addressed. Disease-related information was obtained from electronic medical records or through direct patient interviews. All questionnaire items were mandatory. Surveys that exhibited consistent response patterns, identical answers across all questions, or completion times of less than five minutes were deemed invalid.

### 2.3 Instruments

#### 2.3.1 General information questionnaire

This study was designed by the researchers following an extensive literature review and consisted of two primary components. Demographic characteristics were collected using a self-administered questionnaire that included gender, age, employment status, marital status, place of family residence, education level, monthly household income, medical payment methods, and self-perceived financial stress. Disease-related characteristics included type of disease, duration of diagnosis, total number of recurrence occurrences, current disease status, and IBD-related complications.

In the context of ulcerative colitis (UC), disease activity was assessed using the Wallemarius Simplified Clinical Colitis Activity Index (SCCAI), where scores below than 3 indicate remission and scores of 3 or higher indicate active disease (42). For Crohn’s disease (CD), the Harvey-Bradshaw Index (HBI) was utilized, with scores below 5 indicating remission and scores of 5 or above indicating active disease (43).

#### 2.3.2 Flourishing Scale

The Flourishing Scale (FS), developed by Diener (44) and localized into Chinese by Lai Qiaozhen (45), comprises eight items: meaning, self-acceptance, interpersonal relationships, helping others, engagement, self-competence, optimism about the future, and respect from others. Each item is assessed on a 7-point Likert scale, which ranges from 1 (“strongly disagree”) to 7 (“strongly agree”). Higher scores reflect greater levels of flourishing. In this study, the Cronbach’s α coefficient of this scale was 0.92.

#### 2.3.3 The Inflammatory Bowel Disease Self-efficacy Scale

The Inflammatory Bowel Disease Self-efficacy Scale (IBD-SES), developed by Keefer (46). and localized into Chinese by Tu Wenjing (47), was utilized to assess patients’ confidence in managing their IBD. This scale comprises 29 items, categorized into four dimensions: managing stress and emotions (9 items), managing medical care (8 items), managing symptoms and disease (7 items), and maintaining remission (5 items). Each item is rated on a 10-point Likert scale, ranging from 1 (“no confidence at all”) to 10 (“absolute confidence”). The total score varies from 29 to 290, with higher scores indicating greater self-efficacy in managing IBD. In this study, the Cronbach’s α coefficients for each dimension of the scale ranged from 0.741 to 0.970, the Cronbach’s alpha coefficient for the IBD-SES was 0.83, suggesting good reliability.

#### 2.3.4 Resilience Scale for Inflammatory Bowel Disease

The Resilience Scale for Inflammatory Bowel Disease (RS-IBD) was developed by Dan Luo (48). This questionnaire comprises of 25 items categorized into six dimensions: self-management on disease (four items), active response to difficulty (six items), positive cognition (five items), emotional regulation (four items), family support (three items) and other patients’ support (three items). A 5-point Likert scale was utilized for scoring, where 0 indicates “never” and 4 indicates “always”. The total score ranges from 0 to 100, with higher scores reflecting enhanced mental resilience. In this study, the Cronbach’s α coefficients for each dimension of the scale ranged from 0.765 to 0.952, the Cronbach’s α coefficient was determined to be 0.89.

#### 2.3.5 Social Support Rating Scale

The Social Support Rating Scale (SSRS), developed by Xiao Shuiyuan (49), is designed to assess the level of social support received by patients. This scale encompasses three dimensions: subjective support, objective support, and support availability, comprising a total of 10 items. Among these, Items 1–4 and 8–10 are scored on a scale from 1 to 4; Item 5 offers four options, scored from 1 (“no support”) to 4 (“full support”); for items 6 and 7, participants received a score of 0 if no support was listed, while any selected option was counted as 1 point per option. The total score ranges from 12 to 66, with higher total scores indicating a greater level of social support. To verify the applicability of this scale to the current sample, we conducted tests for the lower limit effect and the upper limit effect. We employed the conventional method (50) for the tests and the results indicated that there were no significant lower limit effects or upper limit effects observed in this study. Additionally, the Cronbach’s α coefficients for each dimension of the scale ranged from 0.825 to 0.849, demonstrating good internal consistency.

### 2.4 Statistical analysis

Data entry and verification were conducted by two trained personnel to ensure accuracy and consistency. Little’s test for completely random missingness indicated a non-significant result (*p* = 0.101), suggesting that data were missing completely at random and were subsequently removed listwise.

Latent profile analysis was conducted using Mplus 8.3, treating the average score of each item in the flourishing scale as a continuous manifest variable. The initial model began with “C1”, and the number of profiles was incrementally increased until the optimal model fit index was achieved. The fitting indicators for the latent profile analysis model included the Akaike Information Criterion (AIC), Bayesian Information Criterion (BIC), sample-adjusted Bayesian Information Criterion (aBIC), information entropy (Entropy), and the likelihood ratio tests: the Lo-Mendell-Rubin corrected likelihood ratio test (LMRT) and the Bootstrap-based likelihood ratio test (BLRT). Among these, lower values of AIC, BIC, and aBIC indicate a better model fit. An entropy value of ≥ 0.8 signifies that the classification accuracy exceeds 90%, with values closer to 1 indicating greater accuracy. The P values for LMRT and BLRT reaching a significant level suggest that the model with k categories is significantly superior to the model with k-1 categories. Through a comprehensive evaluation of the model fit indices and practical interpretability, the optimal latent profile model was identified.

Data analysis was conducted with SPSS 27.0 software. Given that this study primarily relied on patients’ self-reports, which may introduce common method bias, the Harman single-factor method and the unmeasured latent method factor analysis (ULMC) were employed to assess such biases within the scale items. (51). A variance contribution rate of less than 40% for the first factor indicated that common method bias was not severe. Continuous data that adhered to a normal distribution were reported as mean ± standard deviation. The study sample consisted of 316 participants, which is adequate since a sample size of at least 50 is deemed appropriate for the Kolmogorov-Smirnov test for normality (52). Meanwhile, the homoscedasticity test was conducted using the Levene test. Comparisons among multiple groups were performed using one-way analysis of variance, while count data were expressed as frequencies, percentages, or proportions. For unordered categorical variables, the chi-square test or Fisher’s exact test was utilized for comparisons; for ordered categorical variables, the Kruskal-Wallis H test was applied a variance inflation factor (VIF) of 5 or greater indicated a high degree of multicollinearity (53). *Post-hoc* tests were conducted using Bonferroni method for categorical variables and Scheffe’s test for continuous variables. Following the removal of problematic variables, a logistic regression analysis was conducted. Multivariate logistic regression was employed to identify the influencing factors affecting patients’ flourishing in IBD. A *p*-value of less than 0.05 was considered statistically significant.

### 2.5 Validity and reliability

The study adhered to the STROBE guidelines for reporting observational studies. All participants provided written informed consent in accordance with ethical research requirements. The methods and procedures were designed to comply with established standards for human research and were conducted in strict adherence to relevant regulations and guidelines.

## 3 Results

### 3.1 Participants’ characteristics

In this study, a total of 342 participants were recruited, and 325 valid questionnaires were retrieved, resulting in an effective recovery rate of 95.03%. Little’s MCAR test indicated that the missing data in the 325 valid questionnaires satisfied the assumption of completely missing at random (MCAR). Statistically, the individual-level missing rate was 2.8% (9 cases out of 325), which is below the 5% threshold. Given that the data met the MCAR assumption and the missing rate was low, this study employed the listwise deletion method for handling the missing data (54, 55), which involved removing the 9 individuals with any missing variables. Consequently, a complete dataset of 316 patients was included for statistical analysis (56). (STROBE flow diagram as shown in **Supplementary Figure 1**).

Regarding the demographic characteristics, among the 316 patients with IBD in this study, 63.0% were male and 37.0% were female. The higher proportion of males compared to females aligns with the epidemiological characteristics of IBD in China. According to the Global Burden of Disease Study data from 2019, there is a slight male predominance among Chinese IBD patients, which may be attributed to higher smoking rates among men and differences in hormone levels. In terms of age distribution, the 18–45 age group comprised 56.0%, which is consistent with the core epidemiological characteristics of IBD in young adults. Regarding social roles, 66.8% of the patients were employed, and 80.7% were married. This distribution aligns with the clinical characteristics of IBD patients, who often find themselves in critical periods of both family responsibilities and career development. In terms of clinical features, among the 316 IBD patients, 57.0% had UC and 43.0% had CD. Although the proportion of UC remains higher than that of CD, the two proportions have become closer. The distribution of disease duration predominantly reflects short to medium-term disease courses, with 60.1% having a duration of 1 to less than 5 years. This distribution is related to the rapid increase in the incidence of IBD in China. In terms of disease status, 72.2% of the patients were in a remission period, and 59.2% had no IBD-related complications, which corresponds with the natural course of intermittent attacks and long-term remission typical of chronic inflammatory diseases. Regarding economic burden, 63.0% of the patients experienced moderate economic pressure, while 15.2% faced high economic pressure. This result aligns with the findings regarding the economic burden experienced by patients with IBD and the implementation of medical insurance policies in China. Although infliximab and other biological agents have been included in the 2020 National Medical Insurance Catalogue, the ongoing need for long-term medication and repeated examinations continues to impose significant economic pressure on patients. This situation establishes a crucial clinical foundation for the subsequent analysis of how economic factors affect patients’ psychological states. General information about the 316 IBD patients is summarized in **Table 1**.

**Table 1:** Demographic and clinical characteristics of the participants.

Characteristics	Total (*n* = 316)	Classification of latent classes			*χ^2^/H/F*	*P*
		Class 1 (*n* = 66)	Class 2 (*n* = 182)	Class 3 (*n* = 68)		
Gender					1.186 ^a^	0.553
Male	199(63.0)	44(66.7)	110(60.4)	45(66.2)		
Female	117(37.0)	22(33.3)	72(39.6)	23(33.8)		
Age (years)					0.345 ^b^	0.842
18-<45	177(56.0)	35(53.0)	105(57.7)	37(54.4)		
45-<60	62(19.6)	14(21.2)	33(18.1)	15(22.1)		
≥60	77(24.4)	17(25.8)	44(24.2)	16(23.5)		
Employment status					1.408 ^a^	0.847
Employment	211(66.8)	41(62.1)	125(68.7)	455(66.2)		
Unemployment	58(18.3)	14(21.2)	30(16.5)	14(20.6)		
Student	47(14.9)	11(16.7)	27(14.8)	9(13.2)		
Marital status					13.861 ^a^	<0.001
Married	255(80.7)	43(65.2)	157(86.3) ^d^	55(80.9)		
Unmarried or divorced or widowed	61(19.3)	23(34.8)	25(13.7) ^d^	13(19.1)		
Place of family residence					5.486 ^a^	0.064
A city or town	266(84.2)	57(86.4)	158(86.8)	51(75.0)		
Rural area	50(15.8)	9(13.6)	24(13.2)	17(25.0)		
Education level					0.316 ^b^	0.854
Primary school and below	45(14.2)	9(13.6)	24(13.2)	12(17.6)		
Junior/technical secondary/high school	109(34.5)	24(36.4)	63(34.6)	22(32.4)		
Associate degree or above	162(51.3)	33(50.0)	95(52.2)	34(50.0)		
Monthly household income (yuan)					7.744 ^b^	0.021
<3000	46(14.6)	18(27.3)	22(12.1) ^d^	6(8.8) ^d^		
3000-<5000	204(64.5)	37(56.1)	123(67.6)	44(64.7)		
≥5000	66(20.9)	11(16.7)	37(20.3)	18(26.5)		
Medical payment methods					3.078 ^c^	0.517
The medical insurance for urban residents	265(83.9)	58(87.9)	154(84.6)	53(77.9)		
Rural cooperative medical service	46(14.6)	7(10.6)	25(13.7)	14(20.6)		
Others (at own or public expense)	5(1.5)	1(1.5)	3(1.7)	1(1.5)		
Type of disease					1.023 ^a^	0.610
UC	180(57.0)	41(62.1)	100(54.9)	39(57.4)		
CD	136(43.0)	25(37.9)	82(45.1)	29(42.6)		
Duration of diagnosis (years)					22.624 ^b^	<0.001
<1	28(8.9)	17(25.8)	7(3.8) ^d^	4(5.9) ^d^		
1-<5	190(60.1)	39(59.1)	112(61.6)	39(57.3)		
≥5	98(31.0)	10(15.1)	63(34.6) ^d^	25(36.8) ^d^		
Total number of recurrence occurrences(times)					2.340 ^b^	0.310
<5	26(8.2)	5(7.6)	13(7.1)	8(11.8)		
5-10	249(78.8)	49(74.2)	147(80.8)	53(77.9)		
>10	41(13.0)	12(18.2)	22(12.1)	7(10.3)		
Current disease status					12.713 ^b^	0.002
Remission stage	228(72.2)	39(59.1)	144(79.1) ^d^	45(66.2)		
Mild active stage	62(19.6)	16(24.2)	30(16.5)	16(23.5)		
Moderate to severe active stage	26(8.2)	11(16.7)	8(4.4) ^d^	7(10.3)		
IBD-related complications*					7.406 ^a^	0.025
No	187(59.2)	36(54.5)	101(55.5)	50(73.5) ^e^		
Yes	129(40.8)	30(45.5)	81(44.5)	18(26.5) ^e^		
Self-perceived financial stress					4.976 ^b^	0.083
No stress	69(21.8)	12(18.2)	39(21.4)	18(26.5)		
Moderate stress	199(63.0)	38(57.6)	117(64.3)	44(64.7)		
High stress	48(15.2)	16(24.2)	26(14.3)	6(8.8)		

Data are n (%). Class 1 = low flourishing group, Class 2 = moderate flourishing group, Class 3 = high flourishing group. UC, ulcerative colitis; CD, Crohn disease. *Complications refer to the occurrence of one or more conditions such as gastrointestinal bleeding, intestinal perforation, and intestinal obstruction.

^a^ χ^2^ value; ^b^ Kruskal - Wallis *H* value; ^c^ Fisher’s exact test.

^d^ Compared with Class 1, *P <* 0.05. ^e^ Compared with Class 2, *P* < 0.05.

### 3.2 Tests for common method bias and multicollinearity

The Harman single-factor test identified 29 factors with eigenvalues exceeding 1, with the first factor contributing 15.5% to the variance. This value is below the 40% threshold, indicating the absence of significant common method bias. Furthermore, this study utilized the ULMC to further assess common method bias. The results revealed that the fit indices for the four-factor measurement model were as follows: CFI = 0.821, TLI = 0.809, SRMR = 0.067, and RMSEA = 0.063. After introducing the method factor, the fit indices for the ULMC model improved to CFI = 0.889, TLI = 0.877, SRMR = 0.054, and RMSEA = 0.051. According to the ULMC method’s judgment criteria: where an increase in CFI and TLI should not exceed 0.1, and a decrease in RMSEA and SRMR should not exceed 0.05 the variations in the fit indices for this study remained below the critical thresholds, indicating that severe common method bias is absent. (55) In the univariate analysis, 14 variables were identified as statistically significant. A multicollinearity test was conducted on these variables, revealing that the tolerance levels (0.581 - 0.996) for each model ere below 1, and the VIF ranged from 1.004 to 1.720, confirming the absence of multicollinearity among these variables and thereby enhancing the overall robustness of the analysis model.

### 3.3 Potential profiles analysis results for IBD patients

LPA was conducted using Mplus 8.3, utilizing the mean scores of each item in the Flourishing Scale as manifest variables. Models with one to four potential latent classes were fitted, and the fitting results are presented in **Table 2**. As the number of latent classes increased, the AIC, BIC, and aBIC exhibited a gradual decrease, while the Entropy values consistently exceeded 0.80 across all models. The three-class model yielded the highest Entropy value (0.921), indicating superior classification accuracy and reduced uncertainty in individual class membership. Results from the LMRT and BLRT demonstrated that the two-class model significantly outperformed the one-class model, and the three-class model significantly outperformed the two-class model. Although the BLRT remained significant for the four-class model, the LMRT did not achieve statistical significance, suggesting that the improvement in model fit from the three-class to the four-class model was not substantial. Furthermore, while the AIC, BIC, and aBIC values of the four-class model were slightly lower than those of the three-class model, the reduction was minimal (approximately 1%). Combined with the non-significant LMRT result, this indicates that the substantive value of the improved fit is limited. Comprehensive comparisons revealed that despite the significant BLRT result for the four-class model, it exhibited non-significant LMRT, a minimal decrease (approximately 1%) in information criteria, and a reduced Entropy value. This suggests that adding a fourth class did not yield substantial improvements in model fit or explanatory value. In contrast, the three-class model demonstrated significant statistical fit, reasonable class distribution, and high classification accuracy. Furthermore, the probabilities of IBD patients being assigned to their respective latent classes were 0.967, 0.969, and 0.974, respectively. Therefore, the three-class model was ultimately selected as the optimal model.

**Table 2:** Latent class model fit comparison.

Model	*AIC*	*BIC*	*aBIC*	*Entropy*	*P-*value		Probability of class
					*LMR*	*BLRT*	
1	7413.477	7473.569	7422.821	–	–	–	–
2	6681.763	6775.657	6696.363	0.898	0.003	<0.001	0.323/0.677
3	6275.805	6403.500	6295.661	0.921	<0.001	<0.001	0.209/0.576/0.215
4	6206.260	6367.765	6231.380	0.849	0.116	<0.001	0.196/0.301/0.339/0.164

AIC, Akaike Information Criterion; BIC, Bayesian Information Criterion; aBIC, adjusted Bayesian Information Criterion; LMR (P), P value for the Lo-Mendell-Rubin; BLRT (P), P value for the Bootstrapped Likelihood Ratio Test.

**Figure 1:**
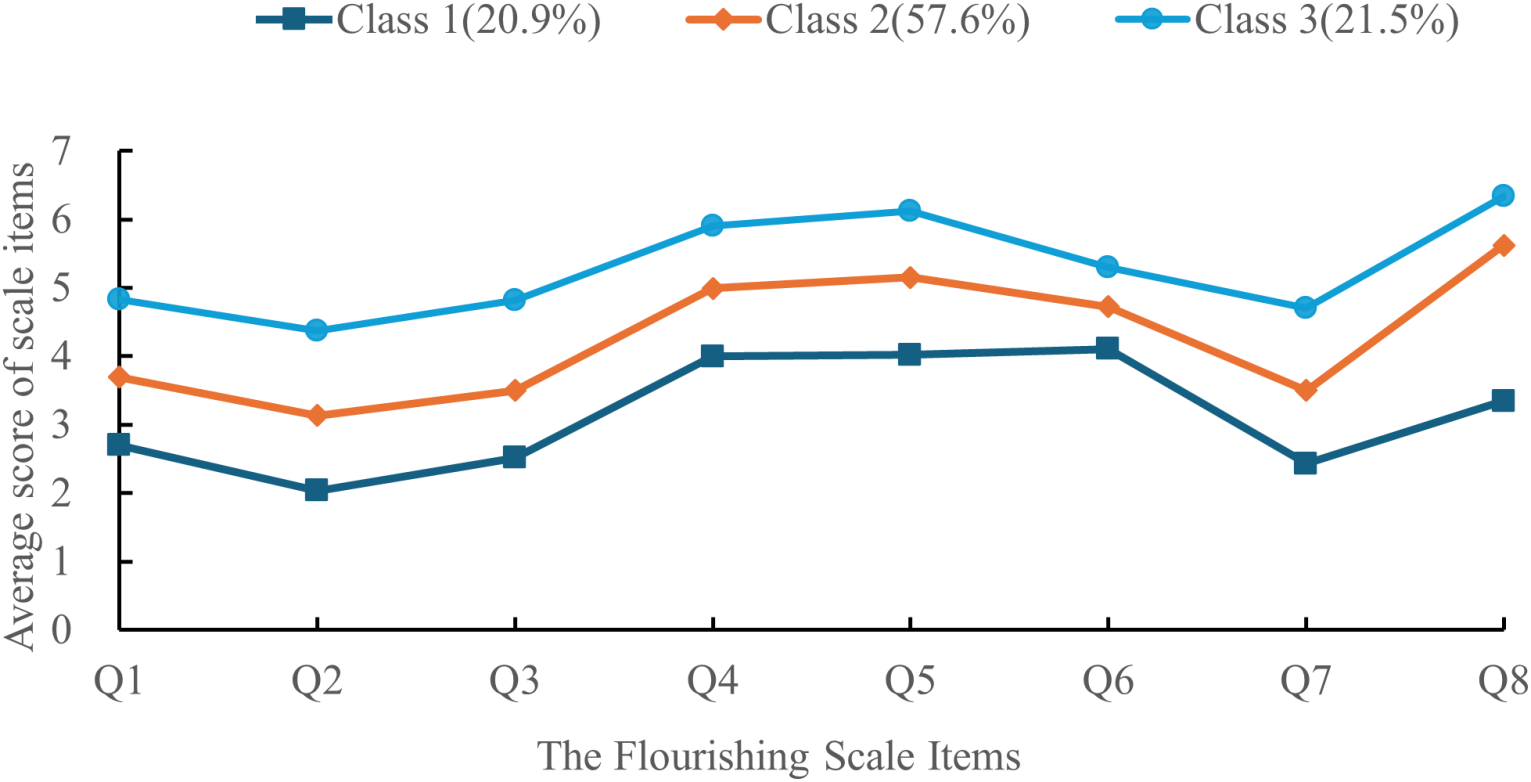
Characteristics of three latent profiles of flourishing in patients with IBD. Class 1 = low flourishing group, Class 2 = moderate flourishing group, Class 3 = high flourishing group.

The flourishing characteristics of patients with IBD are illustrated in **Figure 1**. Classes 1, 2, and 3 are categorized based on their flourishing scale score distributions. Class 1 (n = 66, 20.9%) exhibited notably low average scores across all flourishing scale items, resulting in a total flourishing scale score of 27.82 ± 2.24. Between-group comparisons revealed a mean difference (MD) of -6.66 (95% CI [-7.75, -5.57]) when contrasting Class 1 with Class 2, and -12.82 (95% CI [-14.13, -11.50]) when comparing Class 1 with Class 3. The average scores for individual items ranged from 2.04 to 4.11, reflecting an overall low level of flourishing; thus, this class is designated as the “Low Flourishing Group.” Class 2 (n = 182, 57.6%) demonstrated relatively high average scores for all FS items, culminating in a total flourishing score of 34.34 ± 3.31. Inter-group comparisons indicated an average difference (MD) of -6.16 (95% CI [-7.24, -5.08]) between Class 2 and Class 3, with individual item scores ranging from 3.13 to 5.62. This suggests a moderately elevated level of flourishing, leading to its classification as the “Moderate Flourishing Group.” Class 3 (n = 68, 21.5%) exhibited the highest average scores across all FS items, resulting in a total flourishing score of 40.50 ± 3.49. Individual item scores ranged from 4.38 to 6.34, indicating a favorable state of flourishing; therefore, this class is labeled as the “High Flourishing Group.”

### 3.4 Results of univariate analysis of potential subcategories of IBD patients

The results of the univariate analysis demonstrated statistically significant differences among the three patient categories concerning marital status, monthly household income, duration of diagnosis, current disease condition and IBD-related complications (*p* < 0.05), *Post-hoc* multiple comparisons revealed significant differences in monthly household income (less than 3000) and duration of diagnosis (less than 1 year or greater than or equal to 5 years) between the low flourishing group and both the moderate and high flourishing groups. Additionally, the low flourishing group exhibited significant statistical differences from the moderate flourishing group regarding marital status and current disease status. Notably, the differences between the moderate flourishing group and the high flourishing group were significant only in terms of IBD-related complications, as shown in **Table 1**. The normality test results, specifically the Kolmogorov-Smirnov test, indicated that the scores of the self-efficacy scale, psychological resilience scale, and social support scale, along with their respective dimensions, followed a normal distribution across the three patient groups. Furthermore, the homogeneity of variance test (Levene test) demonstrated that the p-values for each scale and dimension exceeded 0.05, thus confirming the assumption of homogeneity of variance. Comparisons of the total scores and scores for each dimension of the Chinese version of the self-efficacy scale, the total score of the Chinese version of the resilience scale—including its dimensions of family support, positive cognition, and emotion regulation—as well as the total score of the social support rating scale and its dimensions of subjective support and support availability revealed statistically significant differences (*p* < 0.05). *Post-hoc* multiple comparisons indicated that the high flourishing group had significantly higher scores in self-efficacy, psychological resilience, and social support compared to the other two groups, as detailed in **Table 3**.

**Table 3:** Comparison of the scores of the chinese version of the self-efficacy scale, the psychological resilience scale, and the social support rating scale among patients with inflammatory bowel disease across different latent categories.

Item	Total scores (*n* = 316)	Classification of latent classes			Multiple comparisons	*F* -value	*P* -value
		Class 1 (*n* = 66)	Class 2 (*n* = 182)	Class 3 (*n* = 68)			
Total score of IBD Self-Efficacy Scale	213.5 ± 15.86	200.02 ± 8.28	212.65 ± 14.37^f^	228.87 ± 11.71^fj^	Class 3 > Class 2 > Class 1	86.434	<0.001
managing stress and emotions	60.38 ± 8.58	56.17 ± 7.11	59.10 ± 8.07^f^	67.87 ± 6.53^fj^	Class 3 > Class 2 > Class 1	46.113	<0.001
managing medical care	68.54 ± 2.44	68.23 ± 1.53	68.24 ± 2.31	69.65 ± 3.12 ^fj^	Class 3 > Class 1, Class 3 > Class 2	9.389	<0.001
managing symptoms and disease	46.46 ± 5.96	41.50 ± 2.81	46.64 ± 5.58^f^	50.78 ± 5.66 ^fj^	Class 3 > Class 2 > Class 1	54.696	<0.001
maintaining remission	38.13 ± 4.11	34.12 ± 2.03	38.66 ± 3.96^f^	40.57 ± 3.24 ^fj^	Class 3 > Class 2 > Class 1	62.511	<0.001
Total score of the psychological resilience scale	77.09 ± 6.26	75.27 ± 6.71	77.21 ± 6.16	78.53 ± 5.68^f^	Class 3 > Class 1 Class	4.727	0.009
Family support	10.22 ± 0.96	9.76 ± 0.99	10.30 ± 0.93^f^	10.46 ± 0.84^f^	Class 3 > Class 1, Class 2 > Class 1	11.036	<0.001
Other patients’ support	7.19 ± 2.10	7.14 ± 2.27	7.09 ± 2.12	7.49 ± 1.88	–	0.885	0.414
Self-management on disease	13.46 ± 1.24	13.42 ± 1.39	13.45 ± 1.25	13.53 ± 1.06	–	0.139	0.871
Positive cognition	16.17 ± 1.87	15.62 ± 2.12	16.15 ± 1.90^f^	16.78 ± 1.24 ^fj^	Class 3 > Class 2 > Class 1	6.737	0.001
Emotional regulation	12.43 ± 1.73	11.62 ± 1.31	12.69 ± 1.79^f^	12.51 ± 1.72 ^fj^	Class 2 > Class 1, Class 3 > Class 1	9.865	<0.001
Active response to difficulty	17.62 ± 2.15	17.71 ± 2.05	17.53 ± 2.08	17.76 ± 2.44	–	0.381	0.684
Overall social support score	38.40 ± 5.63	34.02 ± 2.92	38.53 ± 5.75^f^	42.31 ± 4.14 ^fj^	Class 3 > Class 2 > Class 1	47.091	<0.001
Objective support	9.24 ± 2.55	9.08 ± 2.51	9.13 ± 2.46	9.72 ± 2.77	–	1.536	0.217
Subjective support	22.34 ± 4.18	21.15 ± 3.07	22.11 ± 4.10	24.12 ± 4.77 ^fj^	Class 3 > Class 1, Class 3 > Class 2	9.597	<0.001
Support availability	7.25 ± 1.47	6.86 ± 1.40	7.19 ± 1.41	7.81 ± 1.55 ^fj^	Class 3 > Class 1, Class 3 > Class 2	7.707	<0.001

Data are *Mean* ± *SD.* Class 1 = low flourishing group, Class 2 = moderate flourishing group, Class 3 = high flourishing group.

^f^ Compared with Class 1, *P <* 0.05. ^j^ Compared with Class 2, *P* < 0.05.

### 3.5 Multivariate analysis results of potential profile categories in patients with IBD

In this study, the three potential profiles of flourishing in patients with IBD as dependent variables, while the statistically significant variables identified through univariate analysis served as independent variables. The results of the parallel line test indicated a *p*-value of less than 0.05, which did not satisfy the assumptions required for the application of the ordinal logistic regression model (*p* > 0.05). Consequently, we selected the unordered logistic regression model for the multivariate analysis. The allocation of independent variables is detailed in **Table 4**. The multivariate analysis revealed that marital status, monthly household income, duration of diagnosis, scores for managing stress and emotions from the self-efficacy scale, scores for managing symptoms and disease, scores for maintaining remission, and scores for emotion regulation dimensions from the resilience scale were all significant predictors of the latent categories of flourishing among participants (all *p* < 0.05) (see **Table 5**).

**Table 4:** Variable coding scheme.

Variable	Assignment
Marital status	
Married	1
Unmarried or divorced or widowed	2
Monthly household income (yuan)	
<3000	1
3000 - < 5000	2
≥ 5000	3
Duration of diagnosis (years)	
<1	1
1 - < 5	2
≥ 5	3
Current disease status	
Remission stage	1
Mild active stage	2
Moderate to severe active stage	3
IBD-related complications	
No	1
Yes	2

**Table 5:** Multivariate analysis of flourishing.

Item	Class 2 vs Class 1				Class 3 vs Class 1			
	*β*	*P*	*OR*	95%*CI*	*β*	*P*	*OR*	95%*CI*
constant term	-13.504	0.066	–	–	-39.805	<0.001	–	–
Marital status								
Married	1.617	0.003	5.040	1.731-14.678	0.933	0.191	2.543	0.628-10.305
Unmarried or divorced or widowed	Ref							
Monthly household income(yuan)								
< 3000	-1.485	0.032	0.226	0.058-0.882	-1.925	0.029	0.146	0.026-0.817
≥ 5000	Ref							
Duration of diagnosis(years)								
< 1	-1.902	0.011	0.149	0.035-0.641	-0.913	0.347	0.401	0.060-2.689
≥ 5	Ref							
managing stress and emotions	0.021	0.687	1.021	0.921-1.133	0.204	<0.001	1.227	1.087-1.385
managing symptoms and disease	0.138	0.008	1.147	1.037-1.270	0.183	0.003	1.201	1.066-1.352
maintaining remission	0.282	<0.001	1.325	1.154-1.522	0.324	<0.001	1.383	1.162-1.646
Emotional regulation	0.448	0.002	1.565	1.182-2.071	0.340	0.048	1.404	1.003-1.965

Pseudo R-Squares=0.635; The likelihood ratio chi-square test (*χ²* = 248.479, *p* < 0.001).

Class 1 = low flourishing group, Class 2 = moderate flourishing group, Class 3 = high flourishing group.

In comparison to patients classified in the low flourishing group, married individuals with IBD exhibited a significantly higher likelihood of being categorized within the moderate flourishing group (OR = 5.040, *p* = 0.003). Furthermore, when compared to both the moderate flourishing group and the high flourishing group, IBD patients with a household per capita monthly income below 3000 demonstrated an increased probability of being classified in the low flourishing group (all *p* < 0.05). Additionally, patients with a disease duration of less than one year were more likely to belong to the low flourishing group compared to those in the moderate flourishing group (OR = 0.149, *p* = 0.011). Moreover, IBD patients who scored higher in managing stress and emotions had an elevated probability of being included in the high flourishing group, in contrast to patients in the low flourishing group (OR = 1.227, *p* < 0.001). Lastly, IBD patients achieving higher scores in managing symptoms and disease, maintaining remission, and emotion regulation were more likely to belong to both the moderate flourishing group and the high flourishing group compared to those in the low flourishing group (all *p* < 0.05), as illustrated in **Table 5**. In addition, among the multiple logistic regression models employed in this study, the pseudo-R² value was found to be 0.635, indicating that the core variables included account for 63.5% of the variation in the latent categories. The likelihood ratio chi-square test (*χ²* = 248.479, *p* < 0.001) and the observed decrease in the -2 log likelihood value both substantiate that the model demonstrates a good fit and effectively captures the relationship between the independent variables and category assignment.

## 4 Discussion

### 4.1 The flourishing of IBD patients can be categorized into three distinct latent profiles

This study identified three distinct latent profiles of flourishing among patients with IBD: a low flourishing group (20.9%), a moderate flourishing group (57.6%), and a high flourishing group (21.5%). These findings underscore the significant heterogeneity in flourishing levels within this population, thereby providing clear and targeted directions for clinical nursing interventions. Patients in the low flourishing group exhibit notable deficits in their perception of social support and a diminished self-recognition of life value. This vulnerability renders them susceptible to disease-related stress and undermines their confidence in adaptive behaviors. These observations are consistent with the findings of Travis, S.et al (57), which highlight the relationship between social support and psychological well-being in patients with chronic diseases. Healthcare providers should prioritize this group by designing tailored interventions. For instance, establishing peer support groups can enhance their social connections, while guiding family members to provide explicit emotional support is crucial. Additionally, initiating low-intensity disease management training can assist them in gradually building confidence in adaptive behaviors. The moderate flourishing group, which constitutes the largest proportion of participants, exhibits moderate overall flourishing but demonstrates a decline in optimism regarding the future. This decrease in optimism significantly affects their long-term adherence to adaptive behaviors. Therefore, healthcare providers should focus on addressing this deficit in optimism by organizing specialized lectures to educate patients about their disease prognosis. Additionally, guiding patients to establish short-term achievable health goals and helping them recognize the link between daily adaptive behaviors and goal attainment can enhance their motivation and adherence (58). Conversely, patients in the high flourishing group display strong social dignity and positive life attitudes; however, they may encounter challenges with adaptive behaviors in specific situations, such as sudden disease recurrence. This observation is consistent with the findings of Trindade, I. A. et al (59). on high-well-being chronic disease patients. To sustain their elevated level of flourishing, healthcare providers should develop personalized and adaptable interventions. For example, creating flexible health schedules that align with their daily routines and teaching practical skills, such as emotional regulation techniques (including deep breathing, mindfulness, and time management strategies), can help mitigate the impact of unforeseen circumstances on their adaptive behaviors.

### 4.2 The impact of marital status on the flourishing of IBD patients

The results of this study indicated that marital status significantly influences the potential profile categories of flourishing in patients with IBD. Specifically, married patients were more likely to be categorized in the moderate flourishing group compared to their counterparts in the low flourishing group. This phenomenon can be attributed to the fact that married individuals typically enjoy more stable emotional companionship and practical support in their daily lives. Spouses can provide timely comfort during periods of illness-related distress, such as exacerbations of symptoms or treatment side effects, thereby helping patients mitigate negative emotions, such as anxiety and helplessness, which may impede flourishing. Furthermore, in terms of disease management, spouses often assist with medication reminders, dietary supervision, and accompany patients to medical appointments. These supports enhance patients’ sense of security and control over their condition, creating a favorable psychological foundation for maintaining moderate levels of flourishing. Conversely, unmarried or divorced patients may experience greater challenges in coping with illness alone, with fewer opportunities to share their physical and emotional burdens, which may contribute to their higher likelihood of being categorized in the low flourishing group. Sim. et al (60) further noted that singles often face greater familial pressure (e.g., marriage expectations), leading to psychological strain that undermines well-being These findings are consistent with prior research indicating the importance of marital relationships in health outcomes (61, 62). Therefore, it is essential to consider the role of marital relationships in promoting the flourishing of patients with IBD. For unmarried or divorced patients who may lack spousal companionship and practical support, healthcare providers can establish peer support groups to facilitate emotional exchange and experience sharing among patients. Additionally, they can connect these patients with social work services to supplement practical assistance in disease management, such as medication reminders and accompaniment to medical appointments.

### 4.3 The impact of economic pressure on the flourishing of IBD patients

The results of this research indicated that, compared to the moderate flourishing group and the high flourishing group, IBD patients with a household monthly income per capita of less than 3000 were more likely to belong to the low flourishing group. aligning with the findings of Yu et al. (63). Given the chronic nature of IBD, lifelong treatment is often necessary; however, the treatment costs for IBD significantly exceed the average income level in China. This financial strain has led 76.1% of patients to either delay seeking medical care or independently reduce their medication due to financial constraints (63). Low treatment adherence can exacerbate disease symptoms, further increasing medical expenditures, intensifying patients’ psychological burden, and consequently reducing their overall well-being. Based on these findings, it is recommended that healthcare providers conduct thorough assessments of patients’ economic pressures. For those facing significant economic challenges, guidance on medical expense reimbursement options should be provided to enhance treatment adherence.

### 4.4 The impact of disease duration on the flourishing of IBD patients

This study also demonstrated that patients with a disease duration of less than one year were more likely to be classified into the low flourishing group compared to those in the moderate flourishing group. This finding indicates that patients with prolonged courses of IBD may exhibit higher levels of flourishing. This result is inconsistent with the conclusion of Qiu Meiqi et al. (64). The discrepancy may be attributed to differences in disease activity distribution between the two studies. In Qiu’s sample, 58.4% of patients with long disease courses were in the moderate-to-severe active stage and experienced complications such as stenosis and fistulas. The persistent inflammatory burden may have directly impaired psychological adaptation. Conversely, in this study, the majority of long-disease-course patients were in the remission stage, where the accumulated coping resources from long-term disease management mitigated the physiological burden. On the contrary, patients in the early stage of IBD may still be in the adaptation phase to the disease; they often confront uncertainties regarding the disease’s progression, treatment outcomes, and long-term impacts on daily life, which can evoke persistent anxiety, stress, or feelings of helplessness. These negative emotions directly undermine key components of flourishing, such as a sense of life meaning, emotional well-being, and interpersonal engagement. In contrast, patients with a longer disease duration may have accumulated more experience in disease management, established effective coping strategies, and gradually adjusted their life plans to accommodate the disease, thereby developing a more stable psychological state and higher levels of flourishing.

Based on these insights, it is recommended that healthcare professionals prioritize targeted psychological support for patients with IBD who have a short disease duration. This support may include providing disease-related education to clarify the chronic yet manageable nature of IBD, assisting patients in understanding the rationale behind treatment regimens, and alleviating anxiety regarding the uncertainties associated with the condition. Additionally, healthcare providers could facilitate connections between newly diagnosed patients and peer support groups consisting of individuals with longer disease durations, enabling them to gain practical disease management experiences and positive coping strategies through interpersonal interactions.

### 4.5 The impact of self-efficacy on the flourishing of IBD patients

The research findings indicated that self-efficacy scores for patients with IBD in our study exhibited distinct variations across the three flourishing profile categories. Specifically, compared to patients in the low flourishing group, those with higher scores in the managing stress and emotions dimension of self-efficacy were more likely to be classified into the high flourishing group. Furthermore, higher scores in both the managing symptoms and disease dimension and the maintaining remission dimension of the self-efficacy scale were positively correlated with higher flourishing levels, and they were more likely to be classified as part of the moderate flourishing group or the high flourishing group. These findings align with the self-efficacy theoretical framework in chronic disease management (65).

Self-efficacy, as a core psychological resource for coping with chronic illnesses, shows differential associations with flourishing across its distinct functional dimensions (46). Higher efficacy in managing stress and emotions enables patients to regulate negative emotions more effectively, thereby preventing emotional distress from undermining their sense of life meaning and interpersonal engagement. For patients with IBD, the efficacy of managing stress and emotions primarily addresses the psychological distress caused by the uncertainty surrounding the disease. IBD is characterized by recurrent symptoms and unpredictable disease progression, which often induce persistent negative emotions such as anxiety, frustration, and fear of flare-ups (66). Patients with low efficacy in managing stress and emotions may find themselves trapped in a state of “emotional paralysis.” They might avoid social interactions due to fear of embarrassment stemming from their symptoms or lose motivation to pursue life goals, patterns that are linked to the social alienation and diminished sense of life meaning observed in the low flourishing group. Notably, research findings indicate that the dimensions of self-efficacy related to managing symptoms and maintaining remission have a more comprehensive impact on flourishing than those related to managing stress and emotions, as they directly address both physical disease control and psychological well-being (67). This underscores the importance of healthcare professionals designing targeted self-efficacy intervention programs for patients with IBD in our study. For instance, structured training sessions could be developed to improve patients’ skills in symptom monitoring and remission maintenance, while mindfulness-based stress reduction workshops could enhance their emotional regulation efficacy (68). Strengthening the dimensions of self-efficacy may represent a promising avenue for fostering positive psychological outcomes in patients with IBD. However, the effectiveness of such interventions necessitates validation through prospective studies. Furthermore, future research should employ longitudinal designs to elucidate the directional relationships between self-efficacy and flourishing, as well as to clarify the potential bidirectional influences between these constructs.

### 4.6 The impact of psychological resilience on the flourishing of IBD patients

The results of this study illustrated that the emotion regulation dimension of psychological resilience was a significant factor influencing the flourishing of patients with IBD in our study. Specifically, patients with higher scores in this dimension were more likely to be categorized into the moderate flourishing group or the high flourishing group. This finding aligns with the research conducted by Mendiolaza et al (69), which highlights the critical role of emotion regulation in adapting to chronic illnesses. As a core component of psychological resilience, emotion regulation encompasses patients’ abilities to recognize, process, and modulate negative emotions triggered by IBD-related stressors. The proficiency level in emotion regulation significantly affects patients’ flourishing status: those with stronger capacities can better mitigate emotional distress from symptom fluctuations, treatment burdens, or future uncertainties, while those with weaker regulation may experience intensified anxiety, frustration, or hopelessness, undermining their sense of life’s meaning, interpersonal connection, and self-worth (70). Furthermore, IBD patients with effective emotion regulation tend to adopt more adaptive coping behaviors which contribute to higher levels of flourishing. These patients are less likely to be overwhelmed by negative emotions and are more inclined to engage in proactive actions that enhance their quality of life and disease control (71). Consequently, the dimension of emotion regulation within psychological resilience plays a pivotal role in shaping the flourishing outcomes of patients with IBD. This underscores the importance of healthcare providers incorporating emotion regulation training into flourishing-focused interventions. However, the effectiveness of such training in facilitating shifts toward moderate or high flourishing levels, as well as improving overall well-being, would require verification through prospective interventional studies.

### 4.7 Limitation

This study has several limitations that warrant acknowledgment. Firstly, as a cross-sectional study, it cannot investigate causal relationships or the cumulative effects among various influencing factors and the latent profile categories of flourishing. Additionally, participants were recruited from a single hospital, which may limit the generalizability of the findings. In addition, the pre-study sample size was relatively small. Despite validation evidence from patients with similar chronic diseases supporting the scale’s applicability, large-sample confirmatory factor analysis and systematic criterion-related validity tests were lacking for the Chinese IBD patient cohort. Future research should expand the sample size to comprehensively verify the scale’s full psychometric properties in this population, thereby providing a more robust methodological basis for assessing their psychological status. Secondly, the influencing factors of flourishing are likely to be complex and diverse, and this study did not comprehensively account for all relevant variables. Future research should focus on systematically exploring the multi-dimensional influencing factors of flourishing in patients with IBD, including negative psychological factors such as anxiety, depression, perceived stress, stigma, and post-traumatic stress disorder, as well as the severity of the disease, as well as potential confounding variables like disease severity, treatment regimens, and surgical history, which may help to more comprehensively reveal the mechanisms underlying the determinants of flourishing levels. Thirdly, it is important to acknowledge the specific limitations of the LPA method employed in this study. Due to sample size and distribution constraints, we were unable to conduct cross-validation or sample-specific validation. Although the total sample size was 316 participants, splitting the dataset would likely have resulted in subgroups that were too small to generate stable or convergent LPA solutions. Furthermore, since this study adopted a cross-sectional design, it was not possible to track the dynamic changes of various latent categories over time, which may render it difficult to fully confirm that these categories represent stable clinical phenotypes. Moreover, this study did not utilize independent clinical indicators or behavioral outcomes for external validation of the potential categories. Future research could potentially adopt a longitudinal design and incorporate independent clinical assessment tools and behavioral indicators to perform external validation. Finally, in direct comparisons between the first category (n = 66) and the third category (n = 68) using multiple regression analyses, the event per variable (EPV) ratio was found to be approximately 9.6. This value is lower than the traditionally recommended threshold, which may indicate a potential risk of model overfitting. Future research should aim to validate these findings by employing a larger sample size or alternatively by utilizing regularization techniques.

## 5 Conclusions

This study utilized LPA to identify three distinct latent profiles of flourishing among IBD patients: a low flourishing group, a moderate flourishing group, and a high flourishing group. These findings underscore the significant heterogeneity in flourishing within this population. Furthermore, the study demonstrated that flourishing among IBD patients is significantly correlated with factors such as marital status, monthly household income, duration of diagnosis, the dimension of self-efficacy related to managing stress and emotions, the dimension related to managing symptoms and disease, the dimension pertaining to maintaining remission, and the emotion regulation dimension of psychological resilience. Healthcare professionals should implement more accurate and targeted intervention measures based on the characteristics and influencing factors of different potential categories, in order to improve the flourishing levels of patients with inflammatory bowel disease.

## Data Availability

The original contributions presented in the study are included in the article/**Supplementary Material**. Further inquiries can be directed to the corresponding author.
