# Supplementary figures and images for: Flourishing and its influencing factor in inflammatory bowel disease patients: a latent profile analysis
Source: Front Psychiatry. 2026 Feb 20;17:1751497. doi: 10.3389/fpsyt.2026.1751497 (PMC12963293; doi:10.3389/fpsyt.2026.1751497)

## Supplementary file 1 STROBE flow diagram


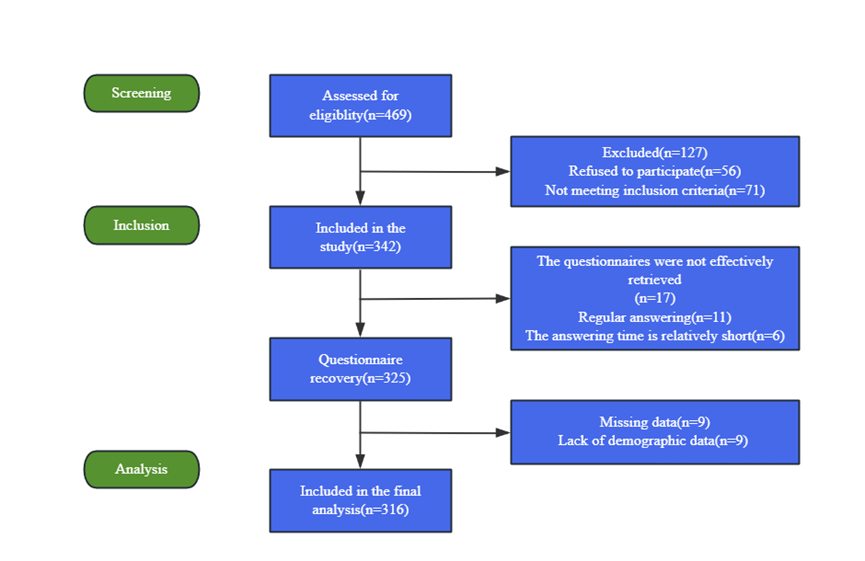

Supplement: Supplementary file 1 [file Supplementaryfile1.docx]
